# Transcriptional profiling of swine mammary gland during the transition from colostrogenesis to lactogenesis using RNA sequencing

**DOI:** 10.1186/s12864-018-4719-5

**Published:** 2018-05-03

**Authors:** V. Palombo, J. J. Loor, M. D’Andrea, M. Vailati-Riboni, K. Shahzad, U. Krogh, P. K. Theil

**Affiliations:** 1 Dipartimento Agricoltura Ambiente e Alimenti, Università degli Studi del Molise, via Francesco De Sanctis s.n.c, 86100 Campobasso, Italy; 2 Department of Animal Sciences, University of Illinois at Urbana-Champaign, Urbana, IL 61801 USA; 3 Department of Animal Science, Aarhus University, Foulum, DK-8830 Tjele, Denmark

**Keywords:** Colostrum, Mammary gland, Sow, Transcriptomics

## Abstract

**Background:**

Colostrum and milk are essential sources of antibodies and nutrients for the neonate, playing a key role in their survival and growth. Slight abnormalities in the timing of colostrogenesis/lactogenesis potentially threaten piglet survival. To further delineate the genes and transcription regulators implicated in the control of the transition from colostrogenesis to lactogenesis, we applied RNA-seq analysis of swine mammary gland tissue from late-gestation to farrowing. Three 2nd parity sows were used for mammary tissue biopsies on days 14, 10, 6 and 2 before (−) parturition and on day 1 after (+) parturition. A total of 15 mRNA libraries were sequenced on a HiSeq2500 (Illumina Inc.). The Dynamic Impact Approach and the Ingenuity Pathway Analysis were used for pathway analysis and gene network analysis, respectively.

**Results:**

A large number of differentially expressed genes were detected very close to parturition (−2d) and at farrowing (+ 1d). The results reflect the extraordinary metabolic changes in the swine mammary gland once it enters into the crucial phases of lactogenesis and underscore a strong transcriptional component in the control of colostrogenesis. There was marked upregulation of genes involved in synthesis of colostrum and main milk components (i.e. proteins, fat, lactose and antimicrobial factors) with a pivotal role of *CSN1S2*, *LALBA*, *WAP*, *SAA2*, and *BTN1A1*. The sustained activation of transcription regulators such as SREBP1 and XBP1 suggested they help coordinate these adaptations.

**Conclusions:**

Overall, the precise timing for the transition from colostrogenesis to lactogenesis in swine mammary gland remains uncharacterized. However, our transcriptomic data support the hypothesis that the transition occurs before parturition. This is likely attributable to upregulation of a wide array of genes including those involved in ‘Protein and Carbohydrate Metabolism’, ‘Immune System’, ‘Lipid Metabolism’, ‘PPAR signaling pathway’ and ‘Prolactin signaling pathway’ along with the activation of transcription regulators controlling lipid synthesis and endoplasmic reticulum biogenesis and stress response.

**Electronic supplementary material:**

The online version of this article (10.1186/s12864-018-4719-5) contains supplementary material, which is available to authorized users.

## Background

Colostrum and milk are essential sources of antibodies and nutrients for the neonate, playing a key role in their survival and growth [1, 2]. In particular, piglet mortality is a major problem especially during the first few days of life [2]. Development of mammary gland is particularly crucial during the final stages of gestation when alveoli begin to distend [3] and there is an abrupt increase in the concentration of colostrum and milk constituents in the swine mammary secretion just prior to parturition [4]. Due to all these rapid developments in such a small time it is clear that any slight abnormalities in colostrogenesis/lactogenesis potentially threaten piglet survival. Hence, characterizing the transcriptome profile and the metabolic and signaling pathways at that stage could provide a more detailed understanding of important molecular mechanisms occurring in the gland during this essential period of reproduction.

Longitudinal transcriptomic studies are ideally-suited for unravelling complex biological behavior at a genome-wide level and provide a more detailed view of the underlying physiological adaptations [5]. In this regard, the development of high-throughput technologies has revolutionized transcriptome analysis. In particular, RNA-Seq technology enables the generation of more extensive transcriptome information providing an advantage over microarray analyses, due to its capability to quantify all transcripts [6].

Recently, RNA-Seq technology has been used in several species to study the lactating mammary gland [7–9]. Although previous studies using microarrays have provided some preliminary insights into the differential expression of genes (DEG) in sow mammary glands around farrowing [10], our understanding of metabolic or signaling pathways in this species is still limited.

The aim of this study was to provide a comprehensive transcriptome profiling of the sow mammary gland from 14 days prior to parturition to day 1 in lactation using RNA Seq analysis and functional bioinformatics tools such as the Dynamic Impact Approach (DIA) [11] and Ingenuity Pathway Analysis (IPA) (Ingenuity Systems, Redwood City, CA).

## Methods

### Animal sampling and RNA extraction

All procedures involving animals were in compliance with Danish laws and regulations for the humane care and use of animals in research [12]. Furthermore, the Danish Animal Experimentation Inspectorate approved the study protocols and supervised the experiment.

Sows used were a subset from an experimental cohort of 36, which involved stratifying animals for body weight at 105 days of gestation to receive one of nine diets (three fiber diets × three fat sources) until day 28 of lactation (weaning) [13]. The test sources of fiber were alfalfa meal or sugar beet pulp with wheat and barley as fiber sources in the control diet. The test fat sources (fed at 30 g/kg dry matter) were soybean oil or glycerol trioctanoate, with palm fatty acid distillate as the fat source in the control. Animals were housed individually in farrowing crates [13]. Mammary tissue collected on days 14, 10, 6 and 2 before (−) parturition and on day 1 after (+) parturition was from three 2nd parity crossbred sows (Danish Landrace × Yorkshire) with the highest colostrum yield (4.6 ± 1.3 kg vs. 2.9 ± 0.9 kg among 9 sows). One of the sows was fed alfalfa meal plus trioctanoate, one sugar beet pulp plus palm fatty acid distillate, and one alfalfa meal plus soybean oil. On the morning of biopsies, sows received only a portion of their meal and upon of milk letdown piglets were removed prior to general anesthesia by intramuscular injection of 1.0 mL/34 kg body weight of Telozol® (Fort Dodge Animal Health, Fort Dodge, IA) dissolved in 2.5 mL ketamine (VetaKet®; Lloyd Laboratories, Shenandoah, IA) and 2.5 mL xylazine-00 (AnaSed®; Lloyd Laboratories). After asceptic surgical prepartion a subcutaneous and intramammary injection of 1.0 mL of 2% lidocaine (Lidoject®; Butler Animal Health supply, Dublin, OH) was given prior to making a 2-cm incision vertical to the plica lateralis. A biopsy consisted of a maximum of three shots using a Manan ProMag 2.2 biopsy gun (Medical Device Technologies, Gainesville, FL) in the same intrusion site while the sow was under anesthesia and in lateral recumbency to expose one entire side of the udder. A total of 20 mg of mammary tissue was collected, after which the incision was closed with simple interrupted sutures [14]. Each sow received 1 mL/100 kg body weight of Banamine (Merck Animal Health, Summit, NJ) immediately after mammary biopsy and at 24 and 48 h post-biopsy. Upon recovery from anesthesia, sows were fed the remainder of their morning meal and piglets were returned to the sow and allowed to suckle normally. Extraction of RNA and quality evaluation was performed following protocols described previously [15]. The average yield of total RNA (from 20.3 ± 6.9 mg tissue) was 44 ± 19 μg, and the average RNA integrity number (Agilent Bioanalyzer) was 8.2 ± 0.8. An aggregate summary of RNA extraction and quality check for all the samples is reported in Additional file 1.

### RNA-sequencing

Sequencing was performed by the High-Throughput Sequencing and Genotyping Unit of the W. M. Keck Biotechnology Center at the University of Illinois at Urbana Champaign (Urbana, IL, USA). A total of 15 mRNA libraries were quantified by qPCR and sequenced on two lanes for 101 cycles from one end of the fragments on a HiSeq2500 (Illumina Inc.), using v4 HiSeq SBS reagents. In total approximately 403 million single-read sequences of 100 nt in length were collected. Quality control metrics were performed on raw sequencing reads using the FASTQC v0.11.15 application. An index of the reference genome was built and single-end clean reads for each individual were aligned to the reference genome using STAR (v2.5.1b). Reads were mapped and annotated to the *Sus scrofa* genome (v10.2.86), downloaded from the EnsemblGenome website (Nov. 2016). Reads aligned were quantified with the Subread package (v1.5.0) based on the Refseq gene annotation.

### Bioinformatics analysis

#### Identification of differentially expressed genes

Non-expressed and weakly expressed genes (i.e. without at least 1 read per million) were removed prior to differential expression (DE) analysis [16]. A TMM (trimmed mean of M-values) normalization was applied to all samples using edgeR [17]. After data were log_2_-transformed, limma-voom method (Bioconductor packages) was used to conduct DE analyses [18, 19]. The applied statistical model included time as fixed effect and animal as random effect. Differentially expressed genes (DEG) across different time points were defined as genes with a Benjamini–Hochberg multiple-testing adjusted *p*-value of ≤0.05. To identify the longitudinal transcriptional gene response close to parturition, the time point − 14 day was used as baseline for each time comparisons. In particular to highlight the metabolic processes underlying mammary changes associated with the colostrogenesis and the onset of lactogenesis in the last stages of gestation leading up to parturition, we relied on DEG between -10vs − 14, −6vs − 14, −2vs-14 and + 1vs-14 time comparisons.

#### Dynamic impact approach (DIA)

The DIA software described previously [11] was used for functional analyses. Briefly, DIA uses the systems information from the KEGG database and ranks pathways calculating the overall impact (importance of a given pathway) and flux (direction of impact; e.g., up-regulation, down-regulation, or no change). For this purpose, the whole dataset (minus weakly expressed genes) with Entrez gene IDs, FDR, FC, and *p*-values of each time comparison were uploaded in DIA and an overall cut-off (FDR and *p*-value ≤0.05) was applied as threshold.

#### Gene network analysis

Ingenuity Pathway Analysis (IPA) was performed to identify transcription regulators and their networks with other genes, within the list of significant DEG (similar cut-off as DIA analysis; FDR and p-value ≤0.05) at each time comparisons (https://www.qiagenbioinformatics.com).

#### Verification by real-time PCR

The expression of *LALBA*, *CSN2*, *PAEP*, and *LTF* was analyzed to verify the physiologic response of the mammary gland as farrowing approached. These genes are well-established markers of mammary-specific genes. Complete information about cDNA synthesis and qPCR performance are reported elsewhere [20]. After normalization with the geometric mean of three internal control genes (*API5*, *VABP*, and *MRPL39*), qPCR data were log_2_-transformed prior to statistical analysis to obtain a normal distribution. Statistical analysis was performed with SAS (v 9.4). Normalized, log_2_-transformed data were subjected to ANOVA with PROC MIXED. The statistical model included time (− 14, − 10, − 6, − 2, and + 1 day from farrowing) as fixed effect, and sow as the random effect. The Kenward-Roger statement was used for computing the denominator degrees of freedom. Fold change for the time comparison -10vs-14, −6vs-14, −2vs-14, and + 1vs-14 were then calculated from the estimates of the model. For each of the four genes and comparison FDR, fold change, and *p*-values are reported in Table 1, together with the respective results from the sequencing analysis.

**Table 1:** Quantitative real time PCR (qPCR) validation of sequencing (Seq) results

		-14 vs −2 time comparison		-14 vs +1 time comparison	
Target	FDR	FC	*P*-value	FC	*P*-value
*CSN2*					
qPCR	<.0001	51.9	<.0001	254.8	<.0001
Seq	0.001	31.0	0.003	33.5	<.0001
*LALBA*					
qPCR	<.0001	156.1	<.0001	8321.9	<.0001
Seq	0.003	128.5	0.03	1275.5	0.001
*LTF*					
qPCR	<.0001	1.5	0.02	3.4	<.0001
Seq	0.001	1.7	0.10	3.9	<.0001
*PAEP*					
qPCR	0.0001	2.2	0.0005	3.4	<.0001
Seq	0.004	1.8	0.01	1.9	0.001

The overall false discovery rate (FDR) together with the fold-change (FC) and P-value for the specific comparison is reported for each gene. The *P*-values were generated applying the same statistical model to either qPCR or Seq data

## Results

### RNAseq analysis and DEG

**Fig. 1:**
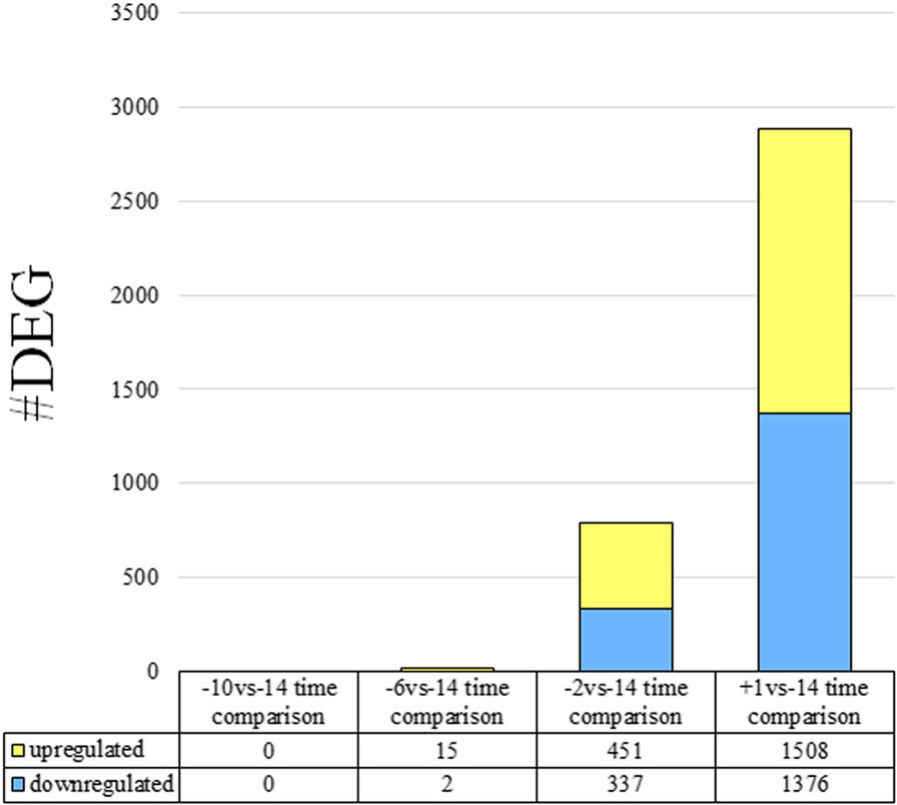
Total number of differentially expressed genes (DEG) due to time resulting from differential expression (DE) analysis of RNAseq data of swine mammary gland tissue harvested in late-pregnancy through farrowing (FDR and p-value ≤0.05). The light blue bars indicate downregulation, while the yellow bars indicate upregulation

An aggregate summary of RNA sequencing and alignment for all the samples is reported in Additional file 2. Illumina sequencing was effective. A large numbers of high-quality reads were produced in all samples. On average, 92% of the total reads were successfully mapped. 91.8% of aligned reads were mapped to unique genomic regions. Results for total number of DEG due to time are presented in Fig. 1. Considering an FDR and p-value ≤0.05 among the 9393 genes (after annotation with the entrez genes ID) a total of 0, 17 (15 upregulated and 2 downregulated), 788 (451 upregulated and 337 downregulated), and 2884 (1508 upregulated and 1376 downregulated) were differentially expressed for -10vs-14, −6vs-14, −2vs-14, +1vs-14 time comparisons, respectively (Additional file 3).

For further analysis with DIA [11] and IPA we focused on DEG between -2vs-14 and + 1vs-14 time comparisons, where the largest numbers of activated and inhibited genes were detected. The -2vs-14 comparison represents the difference in gene expression patterns between a gland with limited growth and a gland that is near full-term, i.e. genes that encompass the last stages of functional differentiation. In contrast, the +1vs-14 comparison represents the difference in mammary tissue between a stage with limited mammary growth and a functional mammary gland which is entered into the lactogenesis stage [4]. To highlight the overall weight of genes in each comparison, the top ten upregulated genes were underscored (Tables 2 and 3). *CSN1S2* and *LALBA* were the most-expressed genes at 2d prepartum, whereas *WAP*, *CSN1S2*, *SAA2* and *LALBA* had a marked upregulation at 1d postpartum. The overlap and specific upregulated genes between the last two time comparisons are reported in Table 4.

**Table 2:** Top ten upregulated genes in the -2vs-14 time comparison (FDR and *p*-value ≤0.05)

Gene symbol	Entrez gene ID	FDR	FC	*P*-value
*CSN1S2*	445,515	˂ .000	629.572	0.002
*LALBA*	397,647	0.003	128.511	0.032
*LOC100522145*	100,522,145	0.002	95.634	0.019
*BTN1A1*	100,153,328	0.007	90.488	0.023
*BTN1A1-like*	100,626,139	0.007	90.488	0.023
*CYP1A1*	403,103	0.005	59.108	0.011
*LOC100524679*	100,524,679	0.006	59.017	0.015
*CSN2*	404,088	0.001	31.014	0.003
*HP*	397,061	˂ .000	29.272	0.001
*ACSL6*	100,522,126	0.012	26.040	0.031

The overall false discovery rate (FDR) together with the fold-change (FC) and P-value for the specific comparison is reported for each gene

**Table 3:** Top ten upregulated genes in the +1vs-14 time comparison (FDR and *p*-value ≤0.05)

Gene symbol	Entrez gene ID	FDR	FC	*P*-value
*WAP*	396,835	0.004	4205.803	0.001
*CSN1S2*	445,515	˂ .000	3858.507	˂ .000
*SAA2*	100,525,680	˂ .000	3039.234	˂ .000
*LALBA*	397,647	0.003	1275.540	0.001
*LOC100522145*	100,522,145	0.002	568.374	˂ .000
*LBP*	397,303	˂ .000	372.186	˂ .000
*CP*	406,870	0.043	258.471	0.011
*HP*	397,061	˂ .000	204.864	˂ .000
*BTN1A1*	100,153,328	0.007	202.517	0.002
*BTN1A1-like*	100,626,139	0.007	202.517	0.002

The overall false discovery rate (FDR) together with the fold-change (FC) and *P*-value for the specific comparison is reported for each gene

**Table 4:** Summary of top 10 upregulated genes in both and specific time comparisons (FDR and *p*-value ≤0.05)

Status	+1vs-14 time comparison	-2vs-14 time comparison	both time comparisons
upregulated	*WAP, SAA2, LBP, CP*	*CYP1A1, LOC100524679, CSN2, ACSL6*	*HP, LALBA, CSN1S2, BTN1A1, BTN1A1-like LOC100522145*

### Overall summary of KEGG categories

**Fig. 2:**
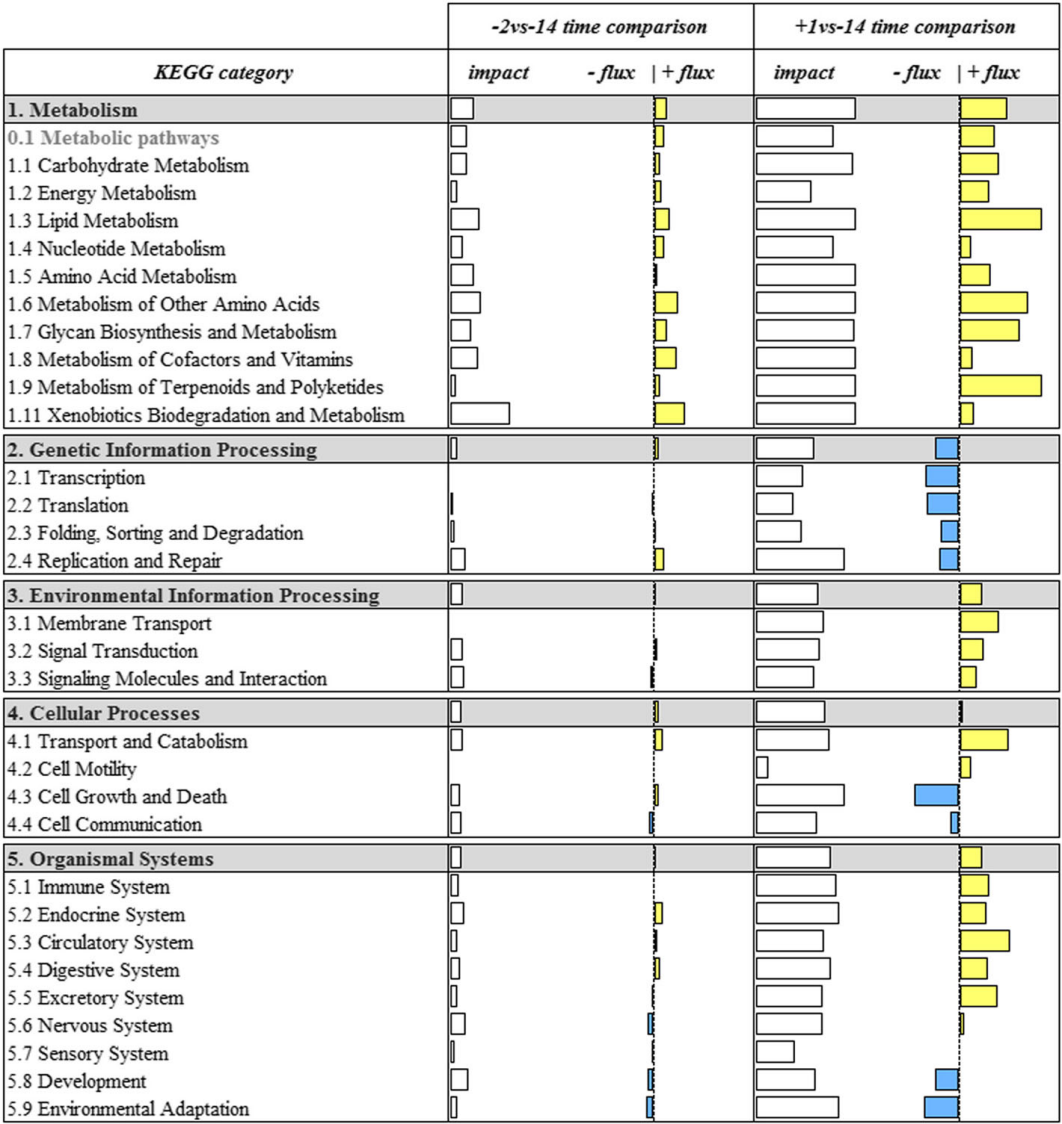
Summary of the main KEGG categories resulting from the Dynamic Impact Approach (DIA) analysis on differentially expressed genes (DEG) obtained by differential expression (DE) analysis of RNAseq data of swine mammary gland tissue harvested in late-pregnancy through farrowing (FDR and *p*-value ≤0.05). For each time comparison, the columns represent the effect (impact) and flux responses. The white bars represent the effect value (0 to 150), and the flux columns represent negative (−) and positive (+) flux (− 150 to + 150) based on the direction of the effect. The negative flux (light blue bars) indicates a downregulation, while the positive flux (yellow bars) indicates an upregulation

The DIA results are summarized in Fig. 2. They provide an overview of impact and flux for each KEGG category calculated following DIA procedures [11]. We clearly observed no significant changes in -10vs-14 and -6vs-14 time comparisons because of the lack of DEG associated with these comparisons (data not showed). Instead, closer to parturition (−2vs-14 comparison), we detected an evident activation of all main categories and in particular ‘Metabolism category’ and ‘Organismal Systems’ pathways, which became stronger considering the postpartum stage (+1vs-14). Focusing only on these main categories and considering the related subcategories with flux value at least 50% of impact value, clearly within ‘Metabolism’ the subcategory ‘Lipid Metabolism’ was the most-impacted and recurrent in both comparisons, followed by ‘Metabolism of Other Amino Acids’. It was not possible to highlight a recurrent subcategory within ‘Organismal Systems’ with flux value at least 50% of impact value in the last 2 comparisons, thus, we chose the recurrent subcategory with highest impact and upregulated flux: ‘Endocrine system’. Regarding the other KEGG pathway categories (i.e. ‘Genetic Information Processing’, ‘Environmental Information Processing’ and ‘Cellular Processes’), we observed a marked downregulation of all ‘Genetic Information Processing’ subcategories and a marked upregulation of ‘Environmental Information Processing’ at 1 day postpartum.

**Fig. 3:**
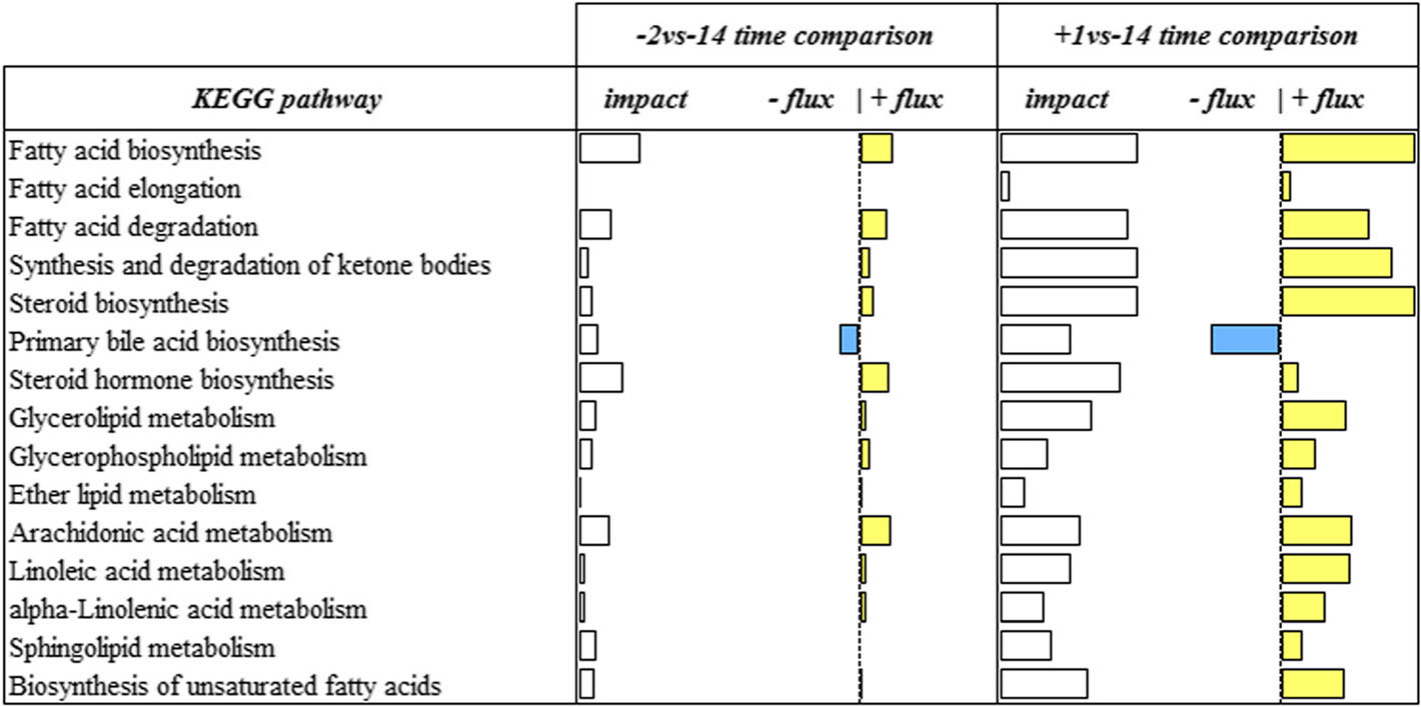
Summary of KEGG ‘Lipid Metabolism’ pathways resulting from the Dynamic Impact Approach (DIA) analysis on differentially expressed genes (DEG) obtained by differential expression (DE) analysis of RNAseq data on swine mammary gland from late pregnancy to farrowing (FDR and p-value ≤0.05). For each time comparison, the columns represent the effect (impact) and flux responses. The white bars represent the effect value (0 to 300), and the flux columns represent negative (−) and positive (+) flux (− 300 to + 300) based on the direction of the effect. The negative flux (light blue bars) indicates a downregulation, while the positive flux (yellow bars) indicates an upregulation

**Fig. 4:**
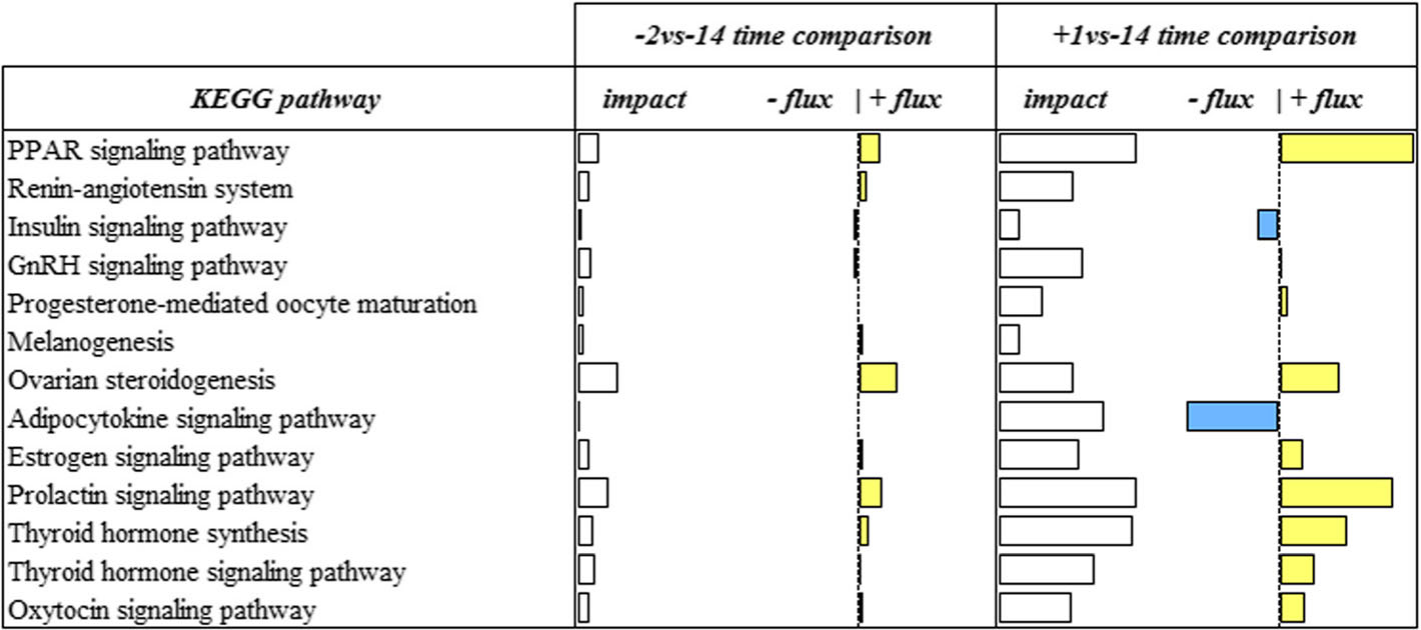
Summary of KEGG ‘Endocrine system’ pathways resulting from the Dynamic Impact Approach (DIA) analysis on differentially expressed genes (DEG) obtained by differential expression (DE) analysis of RNAseq data of swine mammary gland tissue harvested in late-pregnancy through farrowing (FDR and p-value ≤0.05). For each time comparison, the columns represent the effect (impact) and flux responses. The white bars represent the effect value (0 to 200), and the flux columns represent negative (−) and positive (+) flux (−200 to + 200) based on the direction of the effect. The negative flux (light blue bars) indicates a downregulation, while the positive flux (yellow bars) indicates an upregulation

The above general results only provide information about the overall impact and the general direction of the impact (flux) of each significant category and subcategory in the dataset. With the aim to reach a better understanding of the biological relevance of each significant impacted category/subcategories, we focused on the single metabolic pathways falling within the main subcategories of interest particularly ‘Lipid Metabolism’ and ‘Endocrine system’ (Figs. 3 and 4). Except ‘Primary bile acid biosynthesis’, for the ‘Lipid Metabolism’ subcategory we uncovered a clear upregulation of all pathways. In particular, ‘Fatty acid biosynthesis’ was the most impacted and upregulated pathway in both comparisons followed by ‘Arachidonic acid metabolism’ and ‘Steroid hormone biosynthesis’ at 2d prepartum, and ‘Steroid biosynthesis’ and ‘Synthesis and degradation of ketone bodies’ at 1d postpartum. Within ‘Endocrine system’, we detected a marked upregulation of ‘Prolactin signaling pathway’ recurrent in the last 2 comparisons, followed by ‘Ovarian steroidogenesis’ and ‘PPAR signaling pathway’ at 2d prepartum and ‘PPAR signaling pathway’ and ‘Thyroid hormone synthesis’ at 1d postpartum. There was a marked downregulation of ‘Adipocytokine signaling pathway’ at 1d postpartum and ‘Insulin signaling pathway’ in the last 2 comparisons.

### Most impacted pathways

**Fig. 5:**
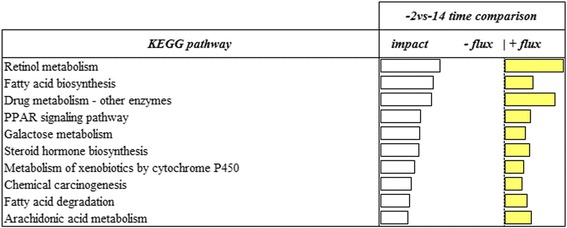
Top 10 upregulated KEGG pathways in the -2d vs −14 d comparison resulting from the Dynamic Impact Approach (DIA) analysis on differentially expressed genes (DEG) obtained by differential expression (DE) analysis of RNAseq data of swine mammary gland tissue harvested in late-pregnancy through farrowing (FDR and p-value ≤0.05). The columns represent the effect (impact) and flux responses. The white bars represent the effect value (0 to 150) and the yellow bars represent the flux (the direction of the effect)

**Fig. 6:**
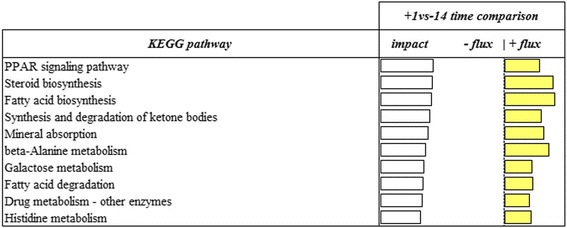
Top 10 upregulated KEGG pathways in the + 1d vs − 14 d comparison resulting from the Dynamic Impact Approach (DIA) analysis on differentially expressed genes (DEG) obtained by differential expression (DE) analysis of RNAseq data of swine mammary gland tissue harvested in late-pregnancy through farrowing (FDR and p-value ≤0.05). The columns represent the effect (impact) and flux responses. The white bars represent the effect value (0 to 400) and the yellow bars represent the flux (the direction of the effect)

To highlight the overall most impacted and upregulated pathways, we considered all pathways without any category classification and with flux value at least 50% of impact value (Figs. 5 and 6). When the last stage (−2d vs -14d) of gestation was considered, ‘Fatty acid biosynthesis’, ‘Retinol metabolism’, ‘Drug metabolism – other enzymes’, ‘PPAR signaling pathway’, ‘Galactose metabolism’, ‘Steroid hormone biosynthesis’, ‘Metabolism of xenobiotics by cytochrome P450’, ‘Chemical carcinogenesis’, ‘Fatty acid degradation’, ‘Arachidonic acid metabolism’ were the most impacted and upregulated pathways. When the parturition stage (+1d vs -14d) was considered, ‘PPAR signaling pathway’, ‘Steroid biosynthesis’, ‘Fatty acid biosynthesis’, ‘Synthesis and degradation of ketone bodies’, ‘Mineral absorption’, ‘beta-Alanine metabolism’, ‘Galactose metabolism’, ‘Fatty acid degradation’, ‘Drug metabolism - other enzyme’ and ‘Histidine metabolism’ were the most impacted and upregulated pathways.

### Enrichment analysis of genes in most recurrent pathway categories

As shown above, ‘Lipid metabolism’ and ‘Endocrine system’ were the most recurrent pathway subcategories. A KEGG enrichment analysis was performed to identify the most upregulated genes among these subcategories (FDR and *p*-value ≤0.05). To underscore the weight of a specific gene within the most recurrent KEGG pathway categories, the genes used by DIA for impact and flux calculations were extrapolated and divided into three groups: upregulated genes in ‘+1vs-14’, ‘-2vs-14’ and ‘both’ time comparisons. The summary of upregulated genes is reported in Table 5.

**Table 5:** Summary of upregulated genes in the most-recurrent KEGG subcategories in both and specific comparisons (FDR and *p*-value ≤0.05)

KEGG Category	Status	+1vs-14 time comparison	-2vs-14 time comparison	Both time comparisons
Lipid Metabolism	upregulated	*ALDH2, HMGCS1, HSD11B1, SCD, ACSL3, FAXDC2, SGMS2, SC5D, 403,334, ACACB, SQLE, MSMO1, CYP2J2, CERS4, CYP2J34, LCLAT1, DHCR24, 100,233,182, PLPP3, FADS2, 100,170,845, 100,517,533, ACAT2, MGLL, CYP2D25, TM7SF2, ACADM, ARSA, PNPLA2, GLA, SMPD1, PTGS1, LPCAT3, GPCPD1, PAFAH2, ACADS, NSDHL, PLA2G12A, KDSR, ECI2, GBA2, COMT, 100515577*	*CYP1A1, 100,157,065, GPAM, GPD2, SGMS1, CDIPT, DGAT1*	*GGT1, 397,097, FADS1, AGPAT1, NEU1, GPAT4, CEPT1, HSD17B7, CDS2, PLA2G16, 100,522,126, 100,522,145, 100,522,692, GPAT3, 100,625,138, 100,625,332, 100,738,292*
Endocrine System	upregulated	*CYP1A1, PYGB*	*396,835, CD14, FXYD2, FOS, HK2, RCAN1, SH2B2, PFKFB2, STAT3, ACACB, PHKG1, ITGB3, HSPA5, OXTR, CYP2J2, PLN, PLCD3, SEC11C, CYP2J34, HSP90B1, MYL9, CPEB4, 100,514,493, ITGA11, RRAS, CREB3L2, CPEB3, CFL2, NFATC2, CTSB, SEC61G, SRP54, SPCS3, PRKCI, ITGA2, PLCD1, JUN, DIAPH1, SEC63, PRKAB2, SP1, BCAR1, B2M, 106,504,143, PRKAB1, PIKFYVE, KAT2B, CREB3, PRKCD, STAT1, RYR2, 100,522,756, 396,848, CASP9, PHKG2, JAK2, ITGAV, SRP68, SRP68, PPP1CB, ITPR3, SOCS6, IFI30, PAK1, PPP1R12C, ELK1, 100,517,270, RHOA*	*CTSV, KCNJ2, NOS3, PCK2, CSN2, FOXO3, MTOR, NCOA2, SPCS1, SRC, SEC61A1, HSD17B7, PDIA3, MAPK14, CALR, SEC61B, RPTOR, SRPRA, CREB3L1, CANX, VAV1, 100,522,176, PDIA4, GNA13, 100,523,015, 100,523,202, EEF2K, MRAS*

### Gene network analysis

IPA allowed uncovering relationships between transcription factors and DEG. Considering a ± 2 ‘Activation z-score’ value and p-value cut-off of 0.01, we identified 6 and 55 upstream transcription regulators (TR) in -2vs-14 and + 1vs-14 time comparisons, respectively (Additional file 4). To highlight and summarize similarities and differences in the activation of TR, the overlap between the two comparisons was performed and the activated TR were extracted. The results are reported in Table 6.

**Table 6:** Summary of the most-activated Transcription Regulators (TR) in both and specific comparisons (*p*-value ≤0.01; z-score ≥ ± 2)

Status	+1vs-14 time comparison	-2vs-14 time comparison	Both time comparisons
Activated	*ATF4, CDKN2A, CEBPA, CREB1, CREM, E2F6, ECSIT, EPAS1, FOXO3, GATA1, HIF1A, ID3, IRF1, IRF3, IRF5, IRF7, KDM5B, MEF2D, MXI1, NFATC2, NFKB1, NFKBIA, NUPR1, PDX1, PPARGC1B, RB1, RBL1, RELA, SMARCA4, SMARCB1, SREBP2, STAT1, STAT2, TCF3, TCF7L2, TOB1, TP53*	*ATF6*	*SREBP1 XBP1*

## Discussion

Although there is some discrepancy in the literature as to the specific timing of colostrogenesis and lactogenesis, the consensus is that swine lactogenesis is activated in late-pregnancy [4, 21]. Lactogenesis is further subdivided in two stages: lactogenesis I, which occurs in late-pregnancy and is linked to the synthesis of early milk components and to final structural mammary gland (MG) differentiation; and lactogenesis II, which is characterized by the onset of abundant milk secretion [21]. It is also accepted that colostrum production takes place during lactogenesis I and that the transition from colostrum to mature milk occurs within 48 h after parturition, influenced by suckling piglets whose effect enhances the rate of fat secretion and accelerates the increase in lactose concentrations [21]. Our results partly confirmed these timings and indirectly suggest that at 14 days prior to parturition (around day 100 of gestation) the MG already entered stage I lactogenesis, hence, the extremely low number of DEG when comparing both − 10, and -6d to -14d; alternatively, the very low number of DEG during those times could have been a reflection of the low activity of the MG. The large numbers (788) of DEG uncovered in the -2dvs-14d time comparison reflects a strong activation of many metabolic processes compatible with the shifting from stage I to stage II of lactogenesis occurring before parturition also in this species [22]. This is consistent with the consideration that MG reached the greatest degree of structural development at that time, and the preparation for copious milk synthesis and secretion had begun [4, 23].

The transition period from a non-lactating to lactating state requires important metabolic changes to enable the shift of nutrient prioritization from body reserves towards the mammary gland for milk production. It is clear that the marked number of DEG (2884) detected at day 1 postpartum vs 14 day prepartum reflects the extraordinary metabolic changes in the swine mammary gland once it fully entered into crucial phases of lactogenesis. A general overview of DIA results confirm this conclusion. In fact, the overall activation of all ‘Metabolic pathways’ and in particular of ‘Lipid Metabolism’, ‘Metabolism of Other Amino Acids’, ‘Carbohydrate Metabolism’ and ‘PPAR signaling pathways’ is compatible with the transition from a non-lactating to lactating state characterized by the expression of genes associated with synthesis of milk components (i.e. milk proteins, fat and lactose). At the same time, the general inhibition of ‘Genetic Information Processing’, the specific inhibition of ‘Cell Growth and Death’ in ‘Cellular Process’ and of ‘Development’ in ‘Organismal System’ is consistent with the fact that the mammary gland has already significantly grown in mass [3].

At farrowing, the MG is fully involved in the accumulation and secretion of colostrum and milk, with their nutritional and immunological proprieties confirmed by the activation of ‘Immune System’, ‘Endocrine System’ and ‘Excretory System’, as well as by the marked upregulation of *OXTR* (oxytocin receptor) [FC = 3.17] [24]. The role of prolactin secretion, which peaks around farrowing and drives the switch from formation and accumulation of colostrum to synthesis and secretion of milk components [25], is supported by the upregulation of ‘Prolactin signaling pathway’. In summary, although not directly compared, our results indicate that the transition from colostrogenesis to lactogenesis occurs between 6 and 2 days before expected parturition. This is likely attributable to upregulation of a wide array of genes including those involved in ‘Protein and Carbohydrate Metabolism’, ‘Immune System’, ‘Lipid Metabolism’, ‘PPAR signaling pathways’ and ‘Prolactin signaling pathway’.

### Protein and carbohydrate metabolism

It is known that concentrations of total protein in sow mammary secretions are highest at parturition [26] The evident upregulation in the last comparison of ‘Amino Acid Metabolism’ and ‘Metabolism of Other Amino Acids’ pathways is consistent with the strong activation of synthesis of the major milk protein during the onset of lactation. These changes in protein concentrations mirror the changes in immunoglobulin content, highly abundant in colostrum with a gradual decrease in milk, and concurrently a lower casein content in colostrum followed by a high increase during the postpartum [26]. While immunoglobulin concentrations are significantly declining and β-lactoglobulin are relatively constant from the colostrum period through lactation, the proportion of casein and α-lactalbumin increases considerably in the postpartum period [26]. These previously reported responses are confirmed in our results by the strong upregulation of *CSN2*, *CSN1S2*, *CSN1S1* and *LALBA*, which are among the overall top upregulated genes in the last 2 comparisons along with the marked upregulation of *WAP* at 1 day postpartum. The upregulation of *LALBA* deserves particular consideration because of its involvement in lactose biosynthesis [27]. Lactose is the major carbohydrate in sow milk and the major osmole in milk, responsible for drawing water into the secretory vesicles [26]. Lactose concentrations are low in colostrum then increase gradually over the first 2 to 3 days of lactation [26]. Our transcriptomic results confirmed this evidence, showing a marked upregulation of ‘Carbohydrate Metabolism’ during the transition from late pregnancy to parturition, driven by upregulation of many genes and particularly by *LALBA*, *B4GALT1* and *HK2* activity all involved in ‘Galactose Metabolism’ pathway.

### Milk caseins

There was a marked upregulation of *CSN1S2* (alpha-S2-casein) at 2d prepartum and 1d postpartum [FC = 629.57 and 3858.51]. Several studies reported *CSN1S2* as one of the most up-regulated caseins increasing in expression during lactation in bovine [28], pig and mouse [29]. It was also found expressed in colostrum and mid lactation milk in goats [30]. The temporal expression pattern of α-casein genes is similar in many species with *CSN1S2* as the most upregulated followed by *CSN1S1*. Our results confirmed this pattern also in pig with a lower expression of *CSN1S1* [FC = 4.00 and 5.25] compared with *CSN1S2*.

An expected result was the marked upregulation of *CSN2* [FC = 31.01 and 33.52], a member of the β-casein family. The principal protein in human milk is the β-casein, which represents the primary source of essential amino acids for a suckling infant. The increases in expression from 2d prepartum to 1d postpartum of *CSN2* was not surprising because it is known that the proportion casein of total milk protein sharply increases by 24 h postpartum [26] and because caseins, together with the whey proteins, represent the highest percentage of milk protein fraction (~ 90%) [26]. In this regard, it is also known that the mRNA abundance of WAP in monogastric appears to be as high or higher than caseins [29]. This is in agreement with our results at 1d postpartum where we found the abrupt upregulation of *WAP* (whey acidic protein) [FC = 4205.80]. Whey acidic protein is the major whey protein in the milk of many species, including the pig where it is secreted at a consistent level throughout lactation [31]. The increase in expression of *WAP* in monogastrics was proportional to *LALBA* [29]. Our result confirmed this relationship [*LALBA*, FC = 128.51 and 1275.53].

It is well established that energy content of the diet and the simultaneous availability of amino acids (AA) affect milk protein content [29]. In this sense, one of the major limitations for milk protein synthesis is represented by transport of AA. A comprehensive literature review on AA transporters in the mammary gland was conducted by Shennan and Boyd [32]. The article describes a list of transporters including those presented in our results. In this regard, the marked upregulation of *SLC7A4* in the last comparison [FC = 32.27] is noteworthy. *SLC7A4* codes for the CAT-4 protein, related to other members of the SLC7 family of cationic amino acid transporters and found highly expressed in swine placental tissue [33]. Although the exact function of the SLC7A4 protein in the context of AA metabolism is not well-known, considering that *SLC7A4* is an important paralog of *SLC7A1*, coding for the CAT-1 protein, which was identified in porcine MG where its abundance increases at early lactation compared with prepartum and it is positively correlated to β-CN and α-LA [34], we speculate that *SLC7A4* in mammary epithelial cells (MEC) could participate in the uptake of leucine (Leu), hence, stimulating protein synthesis through activation of the mTOR cell signaling pathway [35, 36]. In fact, Krogh et al. [37] showed recently that Leu is the most extracted AA by the sow mammary gland in early lactation (d + 3) and in our study, we detected an upregulation [FC = 2.04] at 1d postpartum of another solute carrier family: *SLC7A8*, coding for LAT2, that, together with LAT1, have been proposed to be involved in Leu uptake in the mammary gland [38]. In the same way, insulin signaling, inducing translation via activation of the mTOR pathway, plays an important role in the control of milk protein synthesis [29]. In particular, the insulin effect prevents mTOR inhibition by blocking (via phosphorylation) the main inhibitors of mTOR: the tuberous sclerosis proteins (i.e., TSC1 and TSC2) [29]. In this regard, it was interesting to note the downregulation of *TSC1* at postpartum stage [FC = − 1.24].

### Lactose synthesis

Expression of *LALBA*, encoding α-lactalbumin, was strongly upregulated at 2d prepartum and 1d postpartum [FC = 128.51 and 1275.53]. This is one of the main milk proteins also involved in ‘Carbohydrate Metabolism’ via activation of ‘Galactose metabolism’. In fact, LALBA is part of the lactose synthetase complex that, inside the Golgi, uses glucose and UDP-galactose as substrates for the synthesis of lactose [39]. Our result is in agreement with findings of other studies, where *LALBA* upregulation was detected towards the end of gestation, just before parturition [40, 41]. This result is also consistent with the gradual increase in α-lactalbumin and lactose concentrations in colostrum compared with milk during the first days of lactation [26]. However, if glands are not suckled from 12 h after parturition (i.e. during the colostrum period), expression of *LALBA* is decreased 24 h after parturition in response to lack of colostrum removal [42].

Regarding the lactose synthase enzyme complex, it was noteworthy that in the last 2 comparisons *B4GALT1* was upregulated [FC = 2.40 and 3.27]. The *B4GALT1* gene encodes one of seven beta-1,4-galactosyltransferase (beta4GalT) proteins of the complex and is unique because it participates both in glycoconjugate and lactose biosynthesis [27].

The transport of UDP-galactose into the Golgi is regulated by SLC35A2, which is considered a rate-limiting process in lactose synthesis [43]. The expression of this gene was upregulated in the 2 last comparisons [FC = 2.05 and 2.01]. The marked upregulation of *HK2* [FC = 5.02] at 1d postpartum is important in the context of lactose biosynthesis. In fact, hexokinase (HK) is considered to have a potential controlling step for glucose availability for lactose synthesis [43]. In rodents, *HK2* is detected only after parturition and it was speculated that its presence may lead to an increase in free glucose for lactose synthesis and improved activity of the pentose phosphate shunt to generate reducing equivalents for lipogenesis [44].

### Immune system

The concept that milk, mammary secretions, and the mammary gland have major roles in immune defense has long been proposed [45]. It is well-established that both colostrum and milk proteins have nutritive and immunological functions for the newborn [46]. This is crucial for pigs that have an epitheliochorial placenta impermeable to immunoglobulins (Ig) [1], thus, neonate survival depends upon the passive acquisition of maternal immunity [47]. Immunoglobulins are the primary protein components of colostrum with an immunological function [48]. Immunoglobulin G, in particular, is the major immunoglobulin in sow colostrum and its concentration remains elevated for the initial hours postpartum and then starts to decline consistently [26]. In bovine it is known that specific transport mechanism allows the transfer of a large amount of IgG immunoglobulins from the blood stream across the mammary barrier into colostrum and milk [49]. In pigs it would also appear that colostrum is not a true mammary secretion since 90% of its immunoglobulin content is of serum origin [50].

The transport of immunoglobulins from maternal plasma to colostrum is highly-selective [51] and it is known that FcRn plays an important role in the IgG transport during colostrum formation in several species [52, 53]. In this regard, our results showed no differential expression of *FCGRT* (Fc fragment of IgG receptor and transporter) among time comparisons. Considering the time-window of our experiment, we speculate that this result is consistent with the need for sustained expression of *FCGRT* as a way to support colostrum synthesis and also as a defense mechanism to protect mammary tissue. In fact, it is known that FcRn expression coincides with Stage 1 lactogenesis (the onset of colostrogenesis).

### Antimicrobial components and chemoattractant activity

Antimicrobial proteins naturally present in colostrum and milk have the ability to kill and inhibit a broad spectrum of bacteria [54, 55]. In this regard, the marked upregulation of *HP* (haptoglobin) in the last 2 comparisons [FC = 29.27 and 204.86] was noteworthy. Haptoglobin is an acute-phase protein responsive to inflammation and infection [56] that has already been shown to exert immune modulating functions on the innate and adaptive immune system of the pig [57]. At 1d postpartum there was also a significant upregulation of *LTF* (lactotransferrin) [FC = 3.92]. This gene is a member of the transferrin gene family and is a major iron-binding protein in milk and body secretions with an antimicrobial activity and an important role in the non-specific immune system [45]. Our result is consistent with the fact that lactotransferrin concentrations in swine colostrum at parturition are high and remain elevated through the first days of lactation [26].

Milk is also known to exert a potent chemotactic activity on neutrophils [58]. The prompt recruitment of neutrophils is important for the containment of a number of pathogens at sites of infection, and represents an innate host defenses against microorganisms [59]. In this sense, the role of the chemokine superfamily that encodes secreted proteins involved in immunoregulatory and inflammatory processes must be underscored. Both *CXCL2* and *CXCL10* encode chemokine antimicrobial proteins with a marked upregulation at 1d postpartum [FC = 17.87 and 5.34, respectively]. Bovine colostrum contains all main chemokines (CXCL1, CXCL2 and CXCL3), but concentrations of CXCL2 are generally the lowest and decrease sharply such that it is undetectable in milk after the onset of lactation [58]. From that standpoint, the strong upregulation of *growth-regulated protein homolog gamma* (also known as *CXCL3*) at 1d postpartum [FC = 36.64] is noteworthy. CXCL3/GRO-gamma is involved in the chemokine signaling pathway and (in the absence of inflammation) is considered the major chemotactic factor for neutrophils secreted constitutively into milk [58]. Our results emphasized a major role of *CXCL2* and *CXCL3* in the transition from colostrum to mature milk in swine, probably to help in the prompt recruitment of neutrophils.

We also detected a marked upregulation of *C7* [FC = 7.43] at 1d postpartum. This gene encodes a serum glycoprotein that forms a membrane attack complex together with complement components C5b, C6, C8, and C9 as part of the terminal complement pathway of the innate immune system. In bovine milk, these complement components are found in high concentration in the first 2 days after parturition and then decrease during the following days [60]. In the last 2 time comparisons we also detected the upregulation of *C4A* (Complement C4A) [FC = 2.05 and 5.73], which acts in concert with other complement components to hasten the destruction of pathogens by phagocytes [61]. It is known that milk and colostrum are rich in host-resistance factors, among the others C4 and C3 proactivators [62]. Even in the absence of cognate interactions, the complement system participates in innate immunity providing efficient and rapid protection [63]. The levels of complement fractions C3 and C4 have been studied in the human transition from colostrum to mature milk, where C3 and C4 decrease over lactation with a highest concentration of C3 in colostrum and a highest concentration of C4 in mature milk [63]. Thus, our results confirm a similar trend in the pig.

The marked upregulation of ceruloplasmin (*CP*) [FC = 258.47] was consistent with previous studies with pigs, where expression of *CP* increases in late pregnancy and especially upon lactation, with a correlation between the degree of mammary mRNA expression and the content of milk ceruloplasmin [64]. Although a specific function for CP in the mammary gland is unknown, it may participate in the metabolism of copper [64].

The antimicrobial protein encoded by *LYZ* (lysozyme) was downregulated in the last 2 comparisons [FC = − 3.22 and − 2.66]. Lysozyme has nonspecific antimicrobial activity that is present in many secretions, tissues, and phagocytic cells of mammals. Its role in swine mammary secretions is not yet well elucidated, even though it is thought to contribute to overall antibacterial activity [65]. Krakowski et al. [66] reported lysozyme activity in sow colostrum immediately after parturition, but Chandan et al. [67] did not find lysozyme activity in sow milk.

Proinflammatory cytokines mediate the early local and systemic responses to microbial challenges and may play a key role in development of the neonatal immune system [68]. In this regard, it was also interesting that IPA results showed a pattern of cytokines predicted to be activated: TNF, IFNG, OSM, IL6, IL1B, TNFSF11, IL5, IFNL1, CSF3, TNFSF13B, IL13, IFNB1, IL1A, IFNA2, IFNA1/IFNA13, IL15, IFNL4, THPO, and IFNK. We also detected the upregulation at 1d postpartum of *IL13RA1* (Interleukin 13 Receptor Subunit Alpha 1) [FC = 1.95], *IL15* (Interleukin 15) [FC = 1.57], *IL17RB* (Interleukin 17 Receptor B) [FC = 2.91], *TNFSF13* (Tumor Necrosis Factor Superfamily Member 13) [FC = 2.31], *TNFRSF12A* (TNF Receptor Superfamily Member 12A) [FC = 1.76], and *TNFRSF1A* (TNF Receptor Superfamily Member 1A) [FC = 1.66]. The presence or transfer of these cytokines has not been clearly verified in porcine colostrum or milk. Furthermore, information about the persistence or function of these maternal cytokines in human suckling neonates, as well as in other species, are also limited [68]. The upregulation of *F7* and *F10* (Coagulation Factor VII and X) at 1d postpartum [FC = 6.44 and 5.01] also is noteworthy, because it is known that the coagulation system plays an important role in the innate immune system, particularly in the early host response to infection [69].

There was an abrupt upregulation of *SAA2* (serum amyloid A-2 protein) [FC = 3039.23] at 1d postpartum. This isoform is considered the most predominant member of the *SAA* family expressed in the swine mammary gland [70]. Rodriguez et al. [70] reported that SAA mRNA production in swine increased during lactation and stimulates the neonatal immune response by enhancing the recruitment of mucosal gut B lymphoblasts and potentially influencing Ig concentrations. The authors speculated this confers active and passive protection on neonates and provides local protection for the mammary gland. The upregulation of *CD14* [FC = 24.65] at 1d postpartum was noteworthy. This gene encodes for a surface antigen involved, with other proteins, in the innate immune response to bacterial lipopolysaccharide. Colostrum has high concentrations of soluble CD14 that decrease over time, with the highest concentration detected in “transitional” milk (0 to 4 d postpartum) [71]. Considering the enrichment of sCD14 in colostrum and milk, Filipp et al. [72] speculated it plays a role in actively stimulating the immune system and homeostasis of IgM of the suckling neonate.

The marked upregulation of *SPP1* (also known as *OST*) at 1d postpartum [FC = 14.61] is in agreement with data from RNA isolated from colostrum and mid lactation milk from goats in which it was the most upregulated gene [30]. *SPP1* encodes for the osteopontin protein. The precise role of osteopontin in the mammary gland is still unclear, but it seems to have a role in the modulation of milk protein gene expression, particularly by enhancing the expression of CSN2 [73, 74]. It has also been associated to mammary gland morphogenesis and newborn immunity [75, 76]. Our result seems to suggest a biologic role of this gene during swine lactation but further analyses are required.

### Pathogen recognition

We detected the upregulation of *TLR2* [FC = 4.45 and 7.32] at 2d prepartum and 1d postpartum. This gene encodes a protein member of the Toll-like receptor (TLR) family, playing a pivotal role in pathogen recognition and activation of innate immunity in human [77]. The main bacterial ligands for TLR2 are peptidoglycan and lipoteichoic acid (LTA) of Gram-positive bacteria [78]. *TLR2* is known to be expressed in mammary epithelial cells in bovine, where, after the recognition of specific molecular motifs (i.e. PAMP), determines a rapid and complex innate cascade [79]. We also detected the upregulation at 1d postpartum of *TLR4* [FC = 2.04], the main signaling receptor for most bacterial LPS, part of the outer membrane of Gram-negative bacteria. TLR4 acts as the signal-transducing receptor not only for whole Gram-negative bacteria but also for the fusion protein from respiratory syncytial virus [80].

The marked upregulation of *LBP* in the last 2 comparisons was surprising [FC = 372.18 and 20.89]. The lipopolysaccharide-binding protein (LBP) is one of the most-abundant proteins during infections with Gram-negative bacteria, and is involved in the acute-phase immunologic response. The main function of this protein is to bind bacterial lipopolysaccharides (LPS) expressed on the outer cell wall of bacteria, acting as a carrier for LPS and to help control LPS-dependent monocyte responses [81]. The expression of LBP was demonstrated also in mouse mammary gland early during involution, accompanied by a strong increase in the expression of CD14 protein [82]. Cow colostrum also contains LBP [83] and there is a marked upregulation of *LBP* in sow liver after parturition when animals experience a normal inflammatory state [84]. Our result seems to confirm an important role of LBP in swine mammary gland.

The upregulation of *LY96* (Lymphocyte Antigen 96) [FC = 5.02] also appears biologically-relevant in the context of pathogen recognition. This gene encodes a protein associated with TLR4 on the cell surface and confers responsiveness to LPS, thus, providing a link between the receptor and LPS signaling. It is known that TLR4 cooperates with LY96 and CD14, both of which were upregulated at 1d postpartum and could indicate a response to mediate the innate immune response to bacterial LPS [85, 86]. The upregulation of *ICAM1* at 1d postpartum was significant [FC = 4.42]. This gene encodes a cell surface glycoprotein, typically expressed on endothelial cells and cells of the immune system. The fact that human milk contains substantial amounts of slCAM-1 indicates that it could affect the immune system of the neonate [87]. This gene also could have a similar role in swine colostrum and milk.

### Lipid metabolism

There was an evident activation of all lipid-related pathways very close to the parturition (2d prepartum). This is consistent with the consideration that mammary tissue is preparing to begin copious milk synthesis and secretion. The further upregulation of Lipid Metabolism pathways at 1d postpartum confirmed this and is consistent with the fact that the mammary gland retain fat in late gestation and synthesize great amounts of de novo fat in early lactation [26, 37]. Our results underscored that this transition is likely attributable to upregulation of many genes, including those involved in de novo fatty acid (FA) synthesis, FA activation and desaturation, cholesterol synthesis and ketone body utilization.

### FA de novo synthesis

We observed at 1d postpartum the upregulation of *ACACB* (acetyl-CoA carboxylase-β) [FC = 3.65]. ACC is a complex multifunctional enzyme system, catalyzing the carboxylation of acetyl-CoA to malonyl-CoA, known as the rate-limiting step in fatty acid synthesis. This result is in agreement with expression profile of genes involved in de novo FA synthesis of human mammary gland during secretory activation, where a progressive increase of ACACB activity by day 4 postpartum was highlighted [88]. In contrast, in mouse mammary grand *ACACA* was the only isoform with significant upregulation of expression during lactation, while *ACACB* expression did not differ between pregnancy and lactation [89]. The *ACACA* and *ACACB* encode respectively the isoenzymic ACC proteins: ACCα and ACCβ. The *ACACA* is expressed in the lipogenic tissues and provides cytoplasmic malonyl-CoA for FA synthesis, whereas the *ACACB* is involved in the regulation of β-oxidation of FA in the mitochondria [90]. *FASN* encodes another rate-controlling enzyme in lipogenesis that works in concert with ACACA activity. Both genes play a key role in regulating de novo FA synthesis in bovine mammary gland [90]. However, we did not detect differential expression of *FASN* or *ACACA* between late-gestation and early lactation. Whether this represents a unique feature of the swine mammary gland will have to be established in future experiments.

### FA desaturation genes

The pivotal enzyme implicated in monounsaturated FA synthesis is stearoyl-CoA desaturase (*SCD*), an important enzyme in the mammary gland, which introduces a double bond in the Δ-9 position of myristoyl-, palmitoyl-, and stearoyl-CoA, primarily [91]. The expression of *SCD* was upregulated at 1d postpartum [FC = 5.99], and appears to be central during milk fat synthesis at the onset of lactation in swine mammary gland. This result is in agreement with expression of desaturases in bovine during lactation [91, 92] but is opposite to data from human mammary epithelial cells where its expression decreased over the first 3 days and then gradually increased by day 21 of lactation [88].

Fatty acid desaturase 1 (*FADS1*) and 2 (*FADS2*) play an important role in the synthesis of very-long-chain FA, adding double bonds at the Δ-5 and Δ-6 position of PUFA [93]. FADS1 is involved in the synthesis of the long-chain PUFA arachidonic acid, eicosapentaenoic acid and docosahexaenoic acid. Stage of lactation alters mammary *FADS1* and *FADS2* expression in bovine [91], rat [94, 95], and mouse, with a marked upregulation after parturition. In the latter, FADS1 compared with FADS2 mRNA had a more pronounced and significant upregulation after parturition [89]. Yantao Lv et al. [96] suggested that from late-pregnancy and throughout lactation the swine mammary gland participates in LC-PUFA synthesis by altering the expression of *FADS1* and *FADS2*. The authors speculated that *FADS1* instead of FADS2–3 may play a major role in the biosynthesis of LC-PUFA in the lactating porcine mammary gland. Our results are in agreement with this consideration, since we found *FADS1* gradually increased in the last 2 comparisons (2d prepartum and 1d postpartum) [FC = 2.59 and 4.35], while *FADS2* was significantly activated only in the postpartum [FC = 2.32].

### Glycerol backbone activation

To synthesize triacylglycerol (TAG), both fatty acyl-CoAs and glycerol 3-phosphate must be readily available [97]. The major steps in the pathway of TAG synthesis in mammary gland have been elucidated [98–100]. The activation of the glycerol carbon backbone, which is needed for further acylation, is the first and crucial step for further TAG assembly, and enzymes encoded by glycerol kinase (*GK*) (Ensembl:ENSSSCG00000012202; Additional file 3) and diacylglycerol kinase alpha (*DGKA*) (Ensembl:ENSSSCG00000000370; Additional file 3) play an important role. In particular, glycerol can enter the mammary epithelial cells from the plasma to be phosphorylated by GK [88], and in the last comparison we detected the upregulation of *GK* [FC = 2.41]. We also detected a moderate upregulation in expression of *DGKA* in the last 2 time comparisons [FC = 1.42 and 1.75]. DGKA plays an important role in the resynthesis of phosphatidylinositol and phosphorylation of diacylglycerol to phosphatidic acid. This indicates that the activation of the glycerol carbon backbone, which is needed for further acylation, is crucial during the onset of lactation in swine as in human [88].

### FA internalization and activation

Protein-mediated FA uptake and the flip-flop mechanism play a major role compared with passive diffusion of FA across membranes [91]. The main proteins implicated in FA uptake in non-ruminant cells include fatty acid translocase FAT/CD36 (CD36) and fatty acid transport proteins (FATP or SLC27A) [101]. In bovine, *CD36* was associated with mammary fatty acid uptake from the blood after parturition [91]. Our results underscored a strong upregulation of CD36 [FC = 7.47], hence, confirming its pivotal role in swine mammary gland. The strong upregulation of *ACSL6* (acyl-CoA synthetase long-chain family member 6) in the last 2 comparisons [FC = 26.04 and 37.58] confirmed the importance of ACSL family member isoforms for the FA activation during the onset of lactation in swine as in human [88] and bovine [102]. In fact internalized FAs, prior to participating in further metabolism, must be esterified with CoA in the inner face of the plasma membrane via acyl-CoA (ASC) [91]. In this regard, the upregulation of *ACSL3* at 1d postpartum [FC = 5.62] also is noteworthy given results reported by Lv et al. [96]. They reported that ACSL3 is the most abundant isoform in the porcine mammary gland, in contrast to ACSL1 which is the main isoform in lactating bovine [102] and human [88] mammary cells. The authors speculated that in swine mammary ACSL3 channels LCFA mainly towards TAG synthesis during lactation. This consideration was based on the fact that in the rat ACSL3 prefers C16-C20 unsaturated FA [103], which are major constituents of FA in sow milk [96]. The downregulation of *ACSL1* and *ACSL5* at 2 days prepartum [FC = − 1.44 and − 1.84] seems to support the idea of *ACSL3* being more important during lactation.

### Acyltransferases and TG assembly

From late-pregnancy to onset of lactation, we detected the upregulation in the last 2 comparisons of a cluster of genes involved in the first and rate-limiting step in the TAG biosynthesis pathway, i.e. *GPAT3*, *GPAT4* and *AGPAT1* [FC = 2.04, 2.09 and 1.44; 3.13, 4.46 and 1,47 respectively]. GPAT (glycerol-3-phosphate acyltransferase) enzymes, which reside in the endoplasmic reticulum (ER) and mitochondria, catalyze the first committed step in TAG synthesis via the glycerol phosphate pathway [104]. These enzymes add fatty-acyl groups to the sn-1 position of glycerol-3-phosphate, and lead to the production of monoacylglycerols (MAG) [97]. The conversion of lysophosphatidate to phosphatidate via AGPAT, which adds an acyl group to the sn-2 position of the glycerol backbone, represents the second acylation step in the glycerol phosphate pathway [104].

Regarding specific isoforms uncovered in our results, *GPAT3* is a gene with a controversial identity. Current evidence suggests that it has both GPAT and AGPAT activities [104]. Based on its high amino acid similarity to *AGPAT1* and *AGPAT2*, *GPAT4* was initially classified as *AGPAT6*. Recently after the examination of its enzyme activity, it was considered a second ER-localized GPAT and renamed as GPAT4 [104]. Nagle et al. [105] revealed that GPAT4, expressed in cultured cells, can utilize a variety of substrates, including C12:0-, C16:0-, C18:0-, C18:1-, C18:2-, and C20:4-CoA substrates [105]. *AGPAT1*, which was upregulated in the last 2 time comparisons is a well-established AGPAT isoform, with a validated enzyme activity [106] and a preference for C12–16:0, C16:1, C18:2, and C18:3, followed by C18:0, C18:1, and C20:4, but with a poor activity for C20:0 and C24:0 [104]. Interestingly, *AGPAT1* can also catalyze the reverse of the normal AGPAT reaction, that is the ATP-independent acyl-CoA and LPA (lysophosphatidic acid) synthesis from PA (phosphatidic acid) [107]. This activity suggested its possible implication in regulation of the levels of LPA and PA available to act as signaling molecules [104]. *AGPAT1* (1-Acylglycerol-3-Phosphate O-Acyltransferase 1) was discovered to have a crucial role also during de novo synthesis of triacylglycerol in bovine mammary gland during lactation [102].

Once synthesized and activated, FAs are esterified to glycerol-3-phosphate to produce TAG [96]. Both *GPAM* and *DGAT1* are responsible for the first and last step of esterification leading to TAG synthesis [91]. *GPAM* (glycerol-3-phosphate acyltransferase, mitochondrial) is a well-known gene, mostly expressed in tissues with high lipogenic activity and plays a key role in phospholipid and TAG biosynthesis [108]. *DGAT1* (diacylglycerol acyltransferase 1) also is a well-characterized gene and catalyzes the esterification of the last FA to diacylglycerol leading to TAG synthesis. In the present study both *GPAM* and *DGAT1* were upregulated only at 2d prepartum [FC = 1.91 and 1.31]. In the case of *DGAT1*, this result is not in agreement with studies in bovine and human, where its upregulation occurred postpartum [88, 91]. This may suggest that *DGAT1* is of minor importance in the overall process of milk fat synthesis in the pig, compared with other genes involved in TAG synthesis. In a recent study, however, a western blot analysis of DGAT1 together with other proteins in porcine mammary tissue confirmed its increase during lactation compared with late-pregnancy [96]. Because our time frame of interest was around colostrogenesis, further protein expression and functional studies during these times would have to be conducted to clarify the importance of *DGAT* in colostrogenesis. The fact remains that DGAT1 is one of many proteins composing the TAG synthesis pathway [109, 110].

Because LPIN proteins act in an interdependent manner to optimize lipid homeostasis in various tissues, it is currently believed that their function and role in glycerolipid synthesis are influenced by intricate functional interactions among various LPIN family members [111, 112]. Lv et al. [96] argued for a major role of *LPIN1* in TAG synthesis in the porcine mammary gland during lactation. In the present study, the upregulation of *LPIN1* at 1d postpartum [FC = 2.26] seems to confirm this argument and is in agreement with other studies in human, mouse, and bovine mammary tissue, where a marked upregulation of LPIN1 during lactation was reported [88, 89, 102].

### Lipid droplet formation in milk

After milk fat globule formation in the ER membrane via incorporation of newly-formed TAG, the globules are transported to the apical membrane and released during milk secretion [113]. Butyrophilin (BTN1A1) and xanthine dehydrogenase (XDH) are well-defined proteins involved in these processes in mammary [90], having a function as structural proteins in milk fat droplets in the lactating mammary gland [114]. Furthermore, the essential role of perilipins in droplet formation is well-known [115]. Our data are consistent with this evidence and support a significant role of all *BTN1A1*, *XDH* and *PLIN5* genes in swine mammary lipid droplet formation. In fact, in the last 2 time comparisons we detected a strong upregulation of *BTN1A1* [FC = 90.49 and 202.51] and at 1d postpartum we detected a marked upregulation of *XDH* [FC = 17.24] and *PLIN5* [FC = 21.42]. This is in agreement with a recent study showing that fat is taken up in substantial amounts by the sow mammary glands in late gestation [37].

### Cholesterol synthesis genes

The shift of nutrients from body stores towards the mammary gland for milk production requires not only the adaptation of glucose and lipid metabolism to the lactating state, but also cholesterol metabolism in particular during early lactation [116]. In our results the upregulation of *HMGCS1*, *FAXDC2*, *NSDHL* at 1d postpartum [FC = 7.63; 4.69 and; 1.50] seemed to confirm this evidence also in swine mammary gland. In particular *HMGCS1*, which is important for the regulation of cholesterol synthesis [117], was markedly upregulated during early lactation compared with late pregnancy in the bovine mammary gland [116].

### Utilization of ketone bodies

On day 2 prepartum and day 1 postpartum, we detected moderate upregulation of *BDH* (3-hydroxybutyrate dehydrogenase), encoding a protein catalyzing the initial steps of BHBA utilization in mitochondria [118]. In human, cytosolic type BDH2 is involved in the utilization of ketone bodies, which can subsequently enter mitochondria and the tricarboxylic acid cycle [119]. In ruminants, previous studies showed that the mammary gland takes up large amounts of BHBA and that the use of BHBA (as 4-carbon units) by mammary cells is primarily for de novo FA synthesis [91, 120]. The moderate upregulation of *BDH2* in the last 2 time comparisons [FC = 1.68; 1.93] suggested that ketone bodies likely are an energy source also for the sow mammary gland.

### Ceramide-synthesis genes in mammary

There was moderate upregulation of *SGMS1* (sphingomyelin synthase 1) in the prepartum [FC = 1.42] and higher upregulation of *SGMS2* (sphingomyelin synthase 2) in the postpartum [FC = 4.37], and we found a concomitant downregulation of *SGMS1* [FC = − 1.35]. Sphingomyelin synthases synthesize sphingomyelin through transfer of the phosphatidyl head group in phosphatidylcholine to the primary hydroxyl group of ceramide. Ceramide is one of the most-studied sphingolipids in nature, and is involved in cell signaling, cell cycle, and regulation of protein transport from ER to Golgi [121]. Sphingomyelin synthesis from ceramide is considered an important step because sphingomyelin constitutes about 25% of the total phospholipids in dairy products, having highly bioactive properties and considered to be functional in food [122]. The upregulation of *SMPD1* (sphingomyelin phosphodiesterase 1) [FC = 1.67] and the simultaneously downregulation of *CERS1* (Ceramide Synthase 1) [FC = − 2.12] at 1d postpartum appears to have a biologic role in the overall process of sphingomyelin metabolism. SMPD1 is involved in the conversion of sphingomyelin to ceramide, whereas CERS1 catalyzes the synthesis of ceramide. Further protein expression and functional studies during the entire lactation should be conducted to clarify the role of sphingolipids with signaling roles and the role of ceramide in swine mammary gland.

The marked upregulation of *CYP4A21* [FC = 568.37], a member of the CYP4A subfamily discovered in pig [124], is noteworthy because the protein possesses taurochenodeoxycholic acid 6α-hydroxylase activity but does not metabolise lauric acid, a common substrate for other CYP4As [123]. The function of CYP4A in vivo is not well clarified but CYP4As are known for hydroxylating of a series of fatty acids, eicosanoids and prostaglandins (PG) [124–126]. The activity of CYP4A21 is still uncharacterized in mammary gland. CYP4A21 is believed to be responsible for formation of hyocholic acid, a bile acid typically found in porcine [127]. Further analysis is required to investigate the role of this gene during the onset of lactation in swine mammary gland.

### Transcription factors

The first step of gene expression is transcription and represents the primary step at which gene expression is controlled. This is accomplished through the recruitment of several transcription factors, having the ability to bind to certain target-sequences of the genes, and promote or suppress transcription according to the stimuli [128]. Considering those transcription regulators in the IPA findings that overlap in the last 2 time comparisons (*p*-value cutoff ≤0.01 and activation z-score ≥ ± 2) the results supported the suggestion that SREBP1 and XBP1 are pivotal in the transition from colostrogenesis to lactogenesis in swine mammary gland. They likely act on regulation of lipid synthesis [129] and morphological mammary development [130], respectively.

### Regulation of lipid biosynthesis

The function of SREBP1 (sterol regulatory element-binding protein 1) is well-established in rodents and it plays a crucial role in the regulation of hepatic cholesterol biosynthesis and FA metabolism, in particular the biosynthesis of fat [131, 132]. Our results show that SREBP1 is important also in the mammary gland for cholesterol biosynthesis and this is consistent with real-time PCR measurements that confirmed the upregulation of SREBP1 during the transition from pregnancy to lactation in murine mammary gland [133]. In non-ruminants, SREBP1 is transported from endoplasmic reticulum membrane, where it resides as an inactive precursor, to the Golgi for proteolytic cleavage (i.e., activation) prior to entering the nucleus where it causes the activation of sterol response element (SRE)-containing genes [90]. The transport step to the Golgi is regulated by sterols via the sterol-sensing protein SCAP (SREBP cleavage activating protein), which was modestly upregulated in the last comparison [FC = 1.26].

It is generally accepted that insulin induced gene (INSIG) 1 and 2 encode proteins which interact with SCAP in an oxysterol-dependent and independent fashion (in non-ruminants) and regulate the responsiveness of SREBP1 and 2 processing via SCAP. However, the precise role of INSIG1, strongly upregulated in our last comparison [FC = 13.22], is controversial. In fact, decreased SREBP activity as a consequence of increased *INSIG1* has been observed in liver [134], but upregulation of *INSIG1* was detected during lactation in bovine mammary gland, positively correlated with the ratio of synthesized/imported FA [91].

Our data support a need of *INSIG1* in controlling the induction of gene expression by SREBP. Therefore, *INSIG1* could play a central role in orchestrating lipid metabolism also in swine mammary tissue during lactation. In this regard, the predicted upregulation of PPARG expression at 1d postpartum (known to be involved in regulation of lipid synthesis in goat and bovine mammary cells [91, 135–137]) is noteworthy [PPARGC1B z-score = 2.06]. A potential role of this nuclear receptor in milk fat synthesis was already postulated in particular in bovine mammary gland, where *INSIG1* was demonstrated to be a PPARG responsive gene [91]. PPARG could represent an important control point of milk fat synthesis, in particular in triacylglycerol synthesis and milk secretion in pig as well as in goat and bovine [136, 137], acting indirectly on SREBP1 protein activity through regulation of *INSIG1* expression and directly on SREBP1.

### Regulation of morphological mammary development

Colostrum and milk synthesis occur in alveolar structures composed of a single layer of MEC encircling a lumen where milk is secreted [129]. In order to become fully functional, MEC acquire a number of cellular characteristics during late pregnancy including the development of an elaborate endoplasmic reticulum (ER) system [138], which is required for the synthesis of secreted proteins but is also the site where fatty acids are assembled into TAG and phospholipids [139]. The transcription factor X-box binding protein 1 (XBP1) has multiple functions. Briefly, it promotes ER biogenesis [140] and is a component of a highly-conserved signaling cascade responsible for restoring homeostasis when the ER is confronted with various stresses, including increased protein synthesis and secretion [141, 142]. XBP1 is also implicated as a positive regulator of both lipogenesis and VLDL (very low density lipoprotein) secretion in hepatocytes [143, 144]. Recently, in murine, it was shown that XBP1 is required for MEC population expansion during lactation, enhancing the development of an elaborate endoplasmic reticulum compartment [130] and playing a central role in the coordination of synthesis and export of products in mammary epithelial cells [145].

All the above evidence is consistent with the suggestion that XBP1 may be dispensable for morphologically mammary development, colostrum and milk synthesis and secretion during late-pregnancy and the onset of lactation in pig. In particular, focusing on the upregulated genes involved in protein processing in endoplasmic reticulum that were detected in the last two comparisons, the significant upregulation of *PDIA4* [FC = 2.42 and 3.11], *PDIA3* [FC = 1.60 and 2.15], *PDIA6* [FC = 1.49 and 2.05] and *CALR* (calreticulin) [FC = 1.63 and 2.11] is noteworthy. *PDIA4*, *PDIA3* and *PDIA6* are genes that encode for specific members of the disulfide isomerase (PDI) family of endoplasmic reticulum (ER) proteins catalyzing protein folding and thiol-disulfide interchange reactions. Calreticulin is a multifunctional protein acting in the lumen of the endoplasmic reticulum as a major Ca(2+)-binding (storage) protein. In MEC, it is not completely clear the role of ER-resident proteins on the folding and the retention of milk proteins. However, calreticulin and PDI have been detected in rat and goat lactating MEC, suggesting that these proteins could be involved in the formation of lipid droplets and raising questions about a possible link between the enzymes involved in protein and lipid synthesis [146].

### Other upregulated transcription regulators

At 1d postpartum, we detected a marked upregulation of IRF7 [z-score = 4.69], TP53 [z-score = 4.25], NUPR1 [z-score = 4.10] and NFATC2 [z-score = 4.09] together with XBP1 and SREBP1 which had the highest z-score value. IRF7 encodes interferon regulatory factor 7, a member of the interferon regulatory transcription factor (IRF) family, it is a key transcriptional regulator of type I interferon (IFN)-dependent immune responses with an important role in the innate immune response against DNA and RNA viruses [147]. It regulates the transcription of type I IFN genes (*IFN-α* and *IFN-β*) and IFN-stimulated genes (*ISG*), which were markedly upregulated [*ISG15*, FC = 2.95], by binding in their promoters to an interferon-stimulated response element (ISRE). TP53 (Tumor Protein P53) is a tumor suppressor involved in several types of human tumors, acting both as a gene-specific transcription factor as well as a specific inhibitor of the transcription of certain genes [148]. Its tumor suppressor activity is implicated in the expression of genes involved in the control of cell cycle, cellular senescence, and apoptosis [149] but recently Munne et al. [150] suggested and demonstrated a role for TP53 in the epithelial-to-mesenchymal transition (EMT) and differentiation of mammary epithelia. NUPR1 is a nuclear protein transcriptional regulator involved in the negative regulation of the cell cycle [151]. Zhou et al. [152] reported a high expression of NUPR1, together with other TR, during lactation compared with pregnancy. This could explain why cell cycle-related genes are more active in pregnancy. NFATC2 (nuclear factor of activated t-cells 2) is a member of the nuclear factor of activated T cells (NFAT) family. Most of the work on NFAT proteins has been related to immune cell activation and its mediators, such as cytokines [153]. The product of this gene is a DNA-binding protein with a REL-homology region (RHR) and an NFAT-homology region (NHR) which is present in the cytosol and is only translocated to the nucleus upon T cell receptor (TCR) stimulation. Once in the nucleus it becomes a member of the nuclear factors of the activated T cells transcription complex with a central role in inducing gene transcription during the immune response [154].

## Conclusions

The transcriptome changes greatly during the last week prepartum and these changes are highly likely involved in coordinating the synthesis of colostrum and main milk components (i.e. protein, fat, lactose and antimicrobial factors) as revealed by influenced pathways. The lipid metabolism pathway changes greatly and some of those adaptations are controlled at least in part via SREBP1 and XBP1, acting on regulation of lipid synthesis and morphological development of the mammary gland. Other transcription regulators including IRF7, TR53, NUPR1 and NFATC2 acting across a wide number of pathways become important at the onset of lactation. Further research will help confirm the functional relevance of the pathways uncovered, and how they influence the transition from colostrum to mature milk during a stage when slight abnormalities may potentially threaten piglet survival. Clearly, milk synthesis requires a myriad of factors beyond transcription of the major proteins involved in the synthesis and secretion of protein, fat, and lactose. Holistically, milk synthesis is the product of complex interactions among several tissues and organs that only an integrative systems-biology approach may help elucidate.

## Additional files

Additional file 1: Provides an aggregate summary of RNA extraction and quality check for all samples. Mammary tissue was collected on days 14, 10, 6 and 2 before (−) parturition and on day 1 after (+) parturition from three 2nd parity sows. Extraction of RNA and quality evaluation was performed following protocols described by Tramontana et al. (2008) [15]. (XLSX 10 kb)

Additional file 2: Provides an aggregate summary of RNA sequencing and alignment for all the samples. mRNA libraries were sequenced on a HiSeq2500 (Illumina Inc.). Quality control metrics were performed on raw sequencing reads using the FASTQC v0.11.15 application. An index of the reference genome was built and single-end clean reads for each individual were aligned to the reference genome by STAR (v2.5.1b). Reads were mapped and annotated to the *Sus scrofa* genome (v10.2.86), downloaded from EnsemblGenome website (Nov. 2016). Reads aligned were quantified with Subread package (v1.5.0) based on the Refseq gene annotation. (XLSX 10 kb)

Additional file 3: Provides the complete list of differentially expressed genes (DEG) resulting from differential expression (DE) analysis of RNAseq data of swine mammary gland tissue harvested in late-pregnancy through farrowing and used for Dynamic Impact Approach (DIA) and Ingenuity Pathway Analysis (IPA) analyses. Moreover, it provides additional discussion points, omitted from the main body of the manuscript. (XLSX 1239 kb)

Additional file 4: Provides complete list of upstream regulator resulting from Ingenuity Pathway Analysis (IPA) analysis. Moreover, it provides additional discussion points, omitted from the main body of the manuscript. (XLSX 20 kb)

## Abbreviations

AA: Amino acids
ACACA: Acetyl-CoA carboxylase alpha
ACACB: Acetyl-CoA carboxylase beta
ACC: Acetyl-CoA carboxylase
ACSL: Acyl-CoA synthetase long-chain
ACSL1: Acyl-CoA synthetase long-chain family member 1
ACSL3: Acyl-CoA synthetase long-chain family member 3
ACSL5: Acyl-CoA synthetase long-chain family member 5
ACSL6: Acyl-CoA synthetase long-chain family member 6
AGPAT: 1-Acylglycerol-3-phosphate O-acyltransferase
AGPAT1: 1-Acylglycerol-3-phosphate O-acyltransferase 1
AGPAT2: 1-Acylglycerol-3-phosphate O-acyltransferase 2
ASC: Amino acid transport system ASC
ATP: Adenosine triphosphate
B4GALT1: Beta-1,4-galactosyltransferase 1
BDH: 3-Hydroxybutyrate dehydrogenase
BDH2: 3-Hydroxybutyrate dehydrogenase 2
BHBA: Beta-hydroxybutyric acid
BTN1A1: Butyrophilin subfamily 1 member A1
C3: Complement component 3
C4a: Complement component 4a
C5b: Complement component 5b
C6: Complement component 6
C7: Complement component 7
C8: Complement component 8
C9: Complement component 9
CAT-4: Cationic amino acid transporter 4
CD14: CD14 molecule
CD36: CD36 molecule
CERS1: Ceramide synthase 1
CP: Ceruloplasmin
CSF3: Colony stimulating factor 3
CSN1S2: Casein alpha-S2
CSN2: Casein Beta
CTGF: Connective Tissue Growth Factor
CXCL1: C-X-C Motif chemokine ligand 1
CXCL10: C-X-C Motif chemokine ligand 10
CXCL2: C-X-C Motif chemokine ligand 2
CXCL3: C-X-C Motif chemokine ligand 3
CYP4A: Cytochrome P450 family 4
CYP4A21: Cytochrome P450 family 8 subfamily B member 1
DE: Differential expression
DEG: Differentially expressed gene
DGAT1: Diacylglycerol O-acyltransferase 1
DGKA: Diacylglycerol kinase alpha
DIA: Dynamic impact approach
EMT: Epithelial-to-mesenchymal transition
ER: Endoplasmic reticulum
F10: Coagulation factor X
F7: Coagulation factor VII
FA: Fatty acid
FADS1: Fatty acid desaturase 1
FADS2: Fatty acid desaturase 2
FASN: Fatty acid synthase
FASTQC: Quality control application for FastQ files
FAT: Fatty acid translocase
FATP: Fatty acid transport protein
FAXDC2: Fatty acid hydroxylase domain containing 2
FC: Fold change
FCGRT: Fc fragment of IgG receptor and transporter
FcRn: Neonatal Fc receptor
FDR: False discovery rate
GK: Glycerol kinase
GPAM: Glycerol-3-phosphate acyltransferase, mitochondrial
GPAT: Glycerol-3-phosphate acyltransferase
GPAT3: Glycerol-3-phosphate acyltransferase 3
GPAT4: Glycerol-3-phosphate acyltransferase 4
GRO: Growth-regulated protein homolog
HK2: Hexokinase 2
HMGCS1: 3-Hydroxy-3-methylglutaryl-CoA synthase 1
HP: Haptoglobin
ICAM1: Intercellular adhesion molecule 1
ID: Identification
IFN: Interferon
IFNA1: Interferon alpha 1
IFNA13: Interferon alpha 13
IFNA2: Interferon alpha 2
IFNB1: Interferon beta 1
IFNG: Interferon gamma
IFNK: Interferon kappa
IFNL1: Interferon lambda 1
IFNL4: Interferon lambda 4
Ig: Immunoglobulin
IL13: Interleukin 13
IL13RA1: Interleukin 13 receptor subunit alpha 1
IL15: Interleukin 15
IL17RB: Interleukin 17 receptor B
IL1A: Interleukin 1 alpha
IL1B: Interleukin 1 beta
IL5: Interleukin 5
IL6: Interleukin 6
INSIG: Insulin induced gene
INSIG1: Insulin induced gene 1
IPA: Ingenuity pathway analysis
IRF7: Interferon regulatory factor 7
ISG15: Interferon-stimulated protein 15 KDa
ISRE: Interferon-stimulated response element
KEGG: Kyoto encyclopedia of genes and genomes
LALBA: Lactalbumin alpha
LAT1: Linker for activation of T-cells family member 1
LAT2: Linker for activation of T-cells family member 2
LBP: Lipopolysaccharide-binding protein
LCFA: Long-chain fatty acid
LC-PUFA: Long-chain polyunsaturated fatty acid
Leu: Leucine
LPA: Lysophosphatidic acid
LPIN: Lipin
LPIN1: Lipin 1
LPS: Lipopolysaccharides
LTA: Lipoteichoic acid
LTF: Lactotransferrin
LY96: Lymphocyte antigen 96
LYZ: Lysozyme
MAG: Monoacylglycerols
MEC: Mammary epithelial cells
MG: Mammary gland
mTOR: Mechanistic target of rapamycin
NFAT: Nuclear factor of activated T-cells
NFATC2: Nuclear factor of activated T-cells 2
NHR: NFAT-Homology Region
NSDHL: NAD(P) Dependent steroid dehydrogenase-like
NUPR1: Nuclear protein 1, transcriptional regulator
OSM: Oncostatin M
OST: Osteopontin
OXTR: Oxytocin receptor
PA: Phosphatidic acid
PAEP: Progestagen associated endometrial protein
PAMP: Pathogen associated molecular pattern
PCR: Polymerase chain reaction
PDI: Protein disulfide isomerase
PDIA3: Protein disulfide isomerase family A member 3
PDIA4: Protein disulfide isomerase family A member 4
PDIA6: Protein disulfide isomerase family A member 6
PG: Prostaglandin
PLIN5: Perilipin 5
PPAR: Peroxisome proliferator-activated receptor
PPARG: Peroxisome proliferator activated receptor gamma
PPARGC1B: PPARG coactivator 1 beta
PUFA: Polyunsaturated fatty acid
RHR: REL-Homology Region
SAA2: Serum amyloid A2
SCAP: SREBP Cleavage activating protein
SCD: Stearoyl-CoA desaturase
SCFA: Short-chain fatty acid
SGMS1: Sphingomyelin synthase 1
SGMS2: Sphingomyelin synthase 2
SLC35A2: Solute carrier family 35 member A2
SLC7A4: Solute carrier family 7 member 4
SLC7A8: Solute carrier family 7 member 8
SMPD1: Sphingomyelin phosphodiesterase 1
SPP1: Secreted phosphoprotein 1
SRE: Sterol response element
SREBP1: Sterol regulatory element binding protein 1
STAR: Spliced transcripts alignment to a reference
TAG: Triacylglycerides
TCR: T Cell receptor
TF: Transcription factor
THPO: Thrombopoietin
TLR: Toll like receptor
TLR2: Toll like receptor 2
TLR4: Toll like receptor 4
TMM: Trimmed mean of M-values
TNF: Tumor necrosis factor
TNFRSF12A: TNF Receptor superfamily member 12A
TNFRSF1A: TNF Receptor superfamily member 1A
TNFSF11: Tumor necrosis factor superfamily member 11
TNFSF13: Tumor necrosis factor superfamily member 13
TNFSF13B: Tumor necrosis factor superfamily member 13b
TP53: Tumor protein P53
TR: Transcription regulator
TSC1: Tuberous sclerosis 1
TSC2: Tuberous dclerosis 2
UDP: Uridine diphosphate
VLDL: Very-low-density lipoprotein
WAP: Whey acidic protein
XBP1: X-box binding protein 1
XDH: Xanthine dehydrogenase
α-LA: Lactalbumin alpha
β-CN: Casein beta
